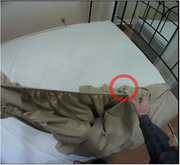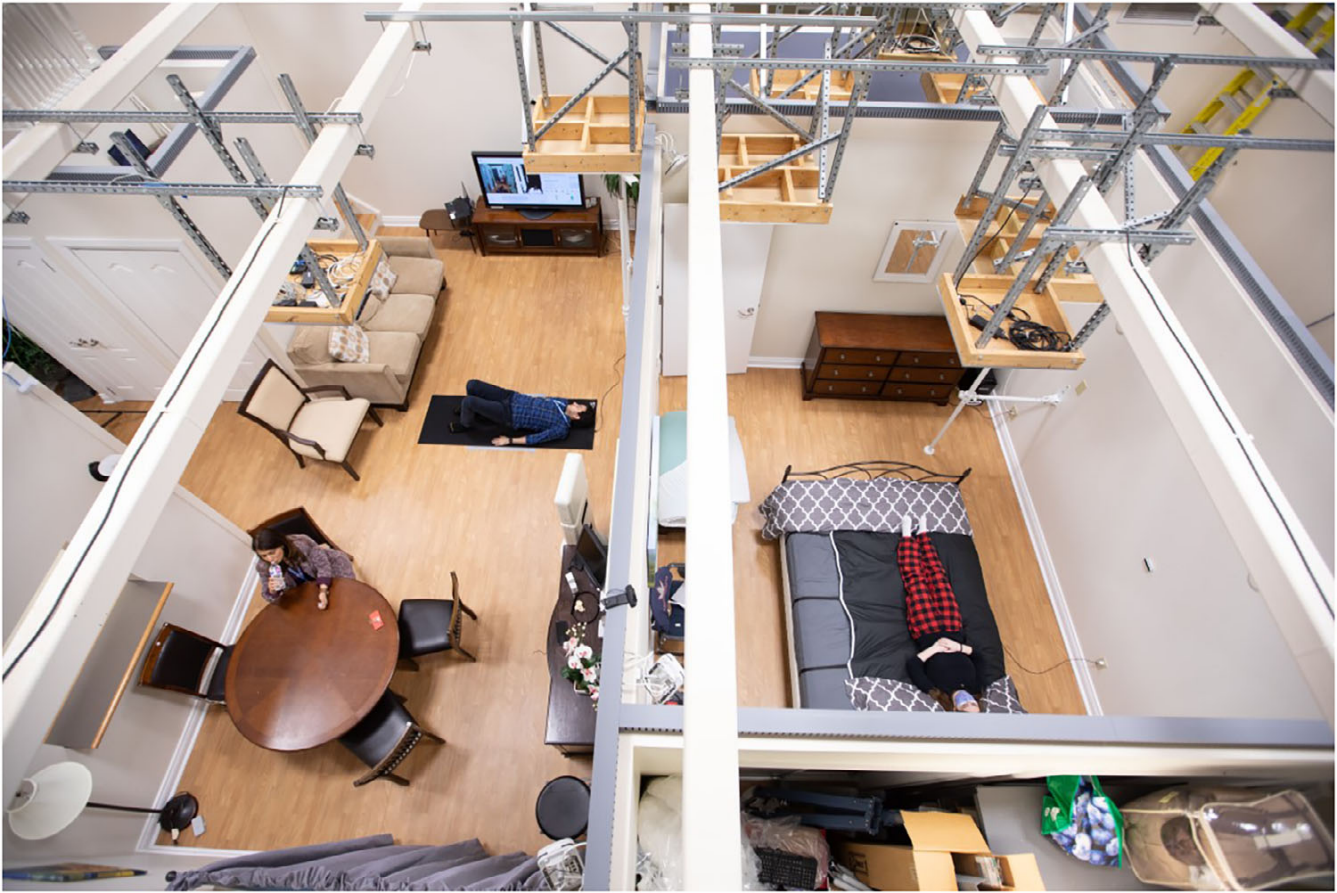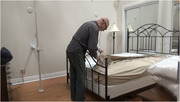# Exploring how individuals with dementia self‐cue while completing three household tasks

**DOI:** 10.1002/alz70858_103066

**Published:** 2025-12-25

**Authors:** Christina Commisso, Shane Avila, Shital Desai, Arlene Astell

**Affiliations:** ^1^ University of Toronto, Toronto, ON, Canada; ^2^ York University, Toronto, ON, Canada; ^3^ Northumbria University, Newcastle upon Tyne, United Kingdom

## Abstract

**Background:**

The decline in ability to complete everyday activities independently is a major determinant of quality of life for people living with dementia. Previous research has shown that saying task‐relevant information out‐loud (‘self‐cueing’) while completing a task assists with children and older adults by helping them to keep on‐task. However, there is limited investigation of the self‐cueing potential in people living with dementia. This study explores how older adults with and without cognitive impairment keep themselves on‐task while completing everyday activities.

**Method:**

Using an observational mixed‐methods study design, thirty older adults with (*n* = 15) and without (*n* = 15) cognitive impairment are completing household tasks in a simulated home environment. Participants are screened using the Montreal Cognitive Assessment (MoCA) tool. Participants in both groups are asked to complete three household tasks in a 1.5‐hour session: (1) make a hot drink, (2) make a bed, and (3) fold laundry. Behaviours are recorded with eye‐tracking glasses, video cameras, a physiological sensor watch, and field‐notes. Survey data regarding task familiarity is also collected. Recordings are thematically analyzed using Noldus Observer XT behavioural analysis software to compare task behaviours between older adults with and without cognitive impairment.

**Result:**

In total, 15 participants without cognitive impairment (MoCA x̄ = 27/30) and 6 participants with cognitive impairment (MoCA x̄ = 21/30) have completed the tasks to date. Behavioural analysis of the video data shows a greater number of interruptions and repetitions during task completion in the cognitive impairment group compared to the control group. Analysis of the eye‐tracking data indicates participants in the control group often look ahead to items involved in the next step of the task while analysis of eye‐tracking data in the cognitive impairment group is currently being explored.

**Conclusion:**

This study is important for understanding how difficulties with self‐cueing may undermine the ability of people living with dementia to stay on‐track during everyday task completion. The findings will be used to inform future interventions aimed at prompting individuals with dementia to stay on‐track for successful task completion. Such interventions could maintain independence, and delay demands on family and services for support with daily activities.